# Comment on “Sarcopenia and cardiovascular diseases: A systematic review and meta‐analysis” by Zuo et al. — The authors reply

**DOI:** 10.1002/jcsm.13389

**Published:** 2023-11-27

**Authors:** Xinrong Zuo, Rui Zhao, Tao Li

**Affiliations:** ^1^ Department of Anesthesiology The Affiliated Hospital of Southwest Medical University Luzhou China; ^2^ Department of Anesthesiology, Laboratory of Mitochondria and Metabolism, National Clinical Research Center for Geriatrics West China Hospital of Sichuan University Chengdu China

We were delighted that our systematic review^1^ aroused people's interest and consideration regarding the association between sarcopenia and cardiovascular diseases (CVDs). Zhang et al.^2^ raised several interesting issues in their letter that we would like to respond to.

First, we rechecked the data and confirmed that our data was correct. In Sasaki's study,^3^ the sample size of sarcopenia in CVDs patients was 8 in males, and 11 in females, respectively, not including sarcopenic obesity and osteosarcopenic obesity. Because sarcopenic obesity is a distinct condition^4^ and obesity is a common risk factor for CVDs, we do not include studies involving sarcopenic obesity in this systematic review. Thus, there are 19 sarcopenia patients in CVDs. As we calculated the prevalence, whichever collection of data was used did not affect the analysis results. However, Zhang et al. extracted incorrect data on CVDs patients (160 people) with sarcopenia (27 people) and calculated the prevalence, so their finding was not precisely correct.

Second, we appreciate Zhang et al.'s suggestion and supplemented the study design. We conducted a subgroup analysis between the study design and the prevalence/incidence of sarcopenia of CVDs patients and calculated the *P* value in Table 1 and concluded that sarcopenia prevalence/incidence was not associated with the study design (Figure 1). In other words, the study design did not affect the increase of incidence or prevalence of sarcopenia in CVDs, which is consistent with our result.

**Table 1:** The prevalence and *p* value of subgroup analysis of study design and diagnostic methods of sarcopenia with CVDs patients

Subgroup	Number of studies	Prevalence	95%CI	*P* value for difference
Cross‐sectional	18	31%	24–38%	0.97
Prospective cohort	4	51%	21–82%	
Comprehensive	11	33%	23–43%	0.21
Non‐comprehensive	11	36%	25–47%	

CI, confidence interval.

**Figure 1:**
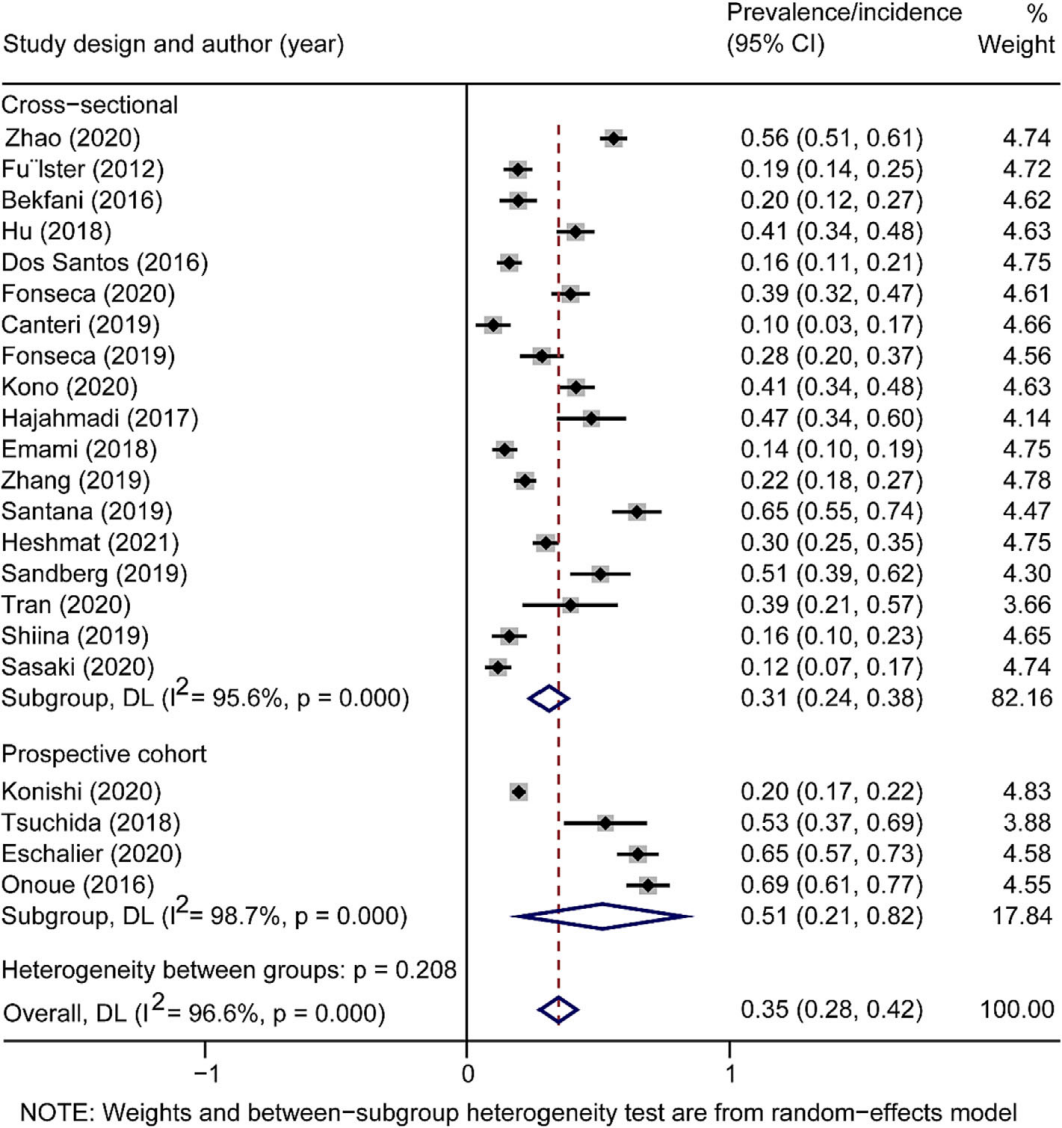
Forest plot of the prevalence of sarcopenia in patients with CVDs of different study designs using a random‐effects model. The *Q* test and *I* ^2^ statistic were used to test the heterogeneity. Note: Weights and between‐subgroup heterogeneity test are from random‐effects model.

Third, in PubMed, Embase, Medline and Web of Science databases, all relevant articles that can be retrieved in English were repeatedly screened and included in our study through strict inclusion and exclusion criteria. Of all the 22 CVDs articles, two were from Chinese journals. Meanwhile, we also did not include relevant articles on the source of the mother tongue in each country.

Fourth, we appreciate Zhang et al.'s supplement.

Fifth, 11 of the selected studies used non‐comprehensive, and 11 studies used comprehensive definitions of sarcopenia. The pooled prevalence of sarcopenia in patients with CVDs was 33% (95% CI: 23–43%) when using the comprehensive criteria and 36% (95% CI: 25–47%) when using the non‐comprehensive criteria, presenting that different definitions of sarcopenia did not cause discrepancy in the prevalence of sarcopenia (*P* = 0.21, Table 1). Further, with the update of the guidelines for sarcopenia, the diagnostic methods for sarcopenia are constantly improving, and different guidelines have different diagnostic efficacy in different populations. In addition, more large multicentre prospective cohort studies are needed to screen the early incidence of sarcopenia and provide a more accurate incidence of sarcopenia and a highly sensitive and specific diagnostic method.

Sixth, we appreciate Zhang et al.'s improvement and conducted meta‐regression of the prevalence by average age. At last, thanks for pointing out the lack of study design, *P* values of different subgroup analyses, and increasing the meta‐regression after the age of homogenization. We have performed a subgroup analysis involving study design, and the corresponding *P* value is calculated.

## Conflict of interest

None declared.

## Funding

None.
